# Protective Role of Helminthiasis in the Development of Autoimmune Diseases

**DOI:** 10.1080/17402520600876895

**Published:** 2006

**Authors:** Francisco Airton Castro da Rocha

**Affiliations:** Laboratory of Investigation of Osteoarthropathies Department of Internal Medicine Federal University of Ceará Fortaleza Brazil


*Clinical & Developmental Immunology*, June–December 2006; 13(2–4): 159–162

Taylor & Francis

Taylor & Francis Group

### Protective role of helminthiasis in the development of autoimmune diseases

FRANCISCO AIRTON CASTRO DA ROCHA

*Laboratory of Investigation of Osteoarthropathies, Department of Internal Medicine, Federal University of Ceará, Fortaleza, Brazil*

**Keywords:** *Helminths, autoimmunity, arthritis, zymosan,* Ascaris, *hyperalgesia*

### Introduction

A role for microorganisms in the development of inflammatory arthropathies has long been recognized. Since the description of reactive arthritis, bacteria have been linked to bouts of arthritis that are thought to occur in a genetically susceptible host, after triggering by an infectious organism.

In a recent article, Toivanen (2003) has proposed that arthritis results from bacteria encountered in the intestinal microbiota. Apparently, processed products of these bacteria gain access to the circulation and are entrapped in the joints, where immunocompetent cells, after being primed by these bacterial products, initiate and may then perpetuate an inflammatory response. Data to support this hypothesis are explored in that paper, as follows: arthritogenic bacteria usually are encountered in the gut. In addition, bacterial products were shown to promote synovitis experimentally and eradication of some infections may ameliorate or even abrogate the development of chronic synovitis. This microbiota hypothesis may also apply to certain viruses. The genetic component that is believed to be relevant in the pathogenesis of chronic arthritis is also contemplated in that "microbiota theory". Indeed, differences in the intestinal flora were reported in patients with rheumatoid arthritis, as compared to healthy individuals. Also, genetic variations may account for differences in the intestinal microbiota. Other examples trying to substantiate this hypothesis are appearing in the literature. In a very recent paper, antibacterial peptide antibodies were proposed to be used as indices to support the diagnosis of rheumatoid arthritis and ankylosing spondylitis (Rashid et al. 2006).

Though we consider this hypothesis very exciting, we will try to explore a somewhat opposite view. The contribution of genetics to the pathogenesis of inflammatory arthropathies cannot be overemphasized. However, to date, despite all the efforts made, the closer and most relevant association of a genetic component for arthritis refers to the human histocompatibility antigen HLA-B27 and the inflammatory spondyloarthropathies (Khan and Ball 2002). However, that association does not apply worldwide, as it is in white Caucasians. Actually, there are reports that some HLA-B27 variants, defined by the most recent molecular biology techniques, may even confer protection for the development of spondylitis (Khan and Ball 2002).

The reduced coincidental prevalence of autoimmune disease among identical twins does also argue against a prominent genetic component in the pathogenesis of these entities. In rheumatoid arthritis, the concordance rate for twins varies from 15 to 30% (MacGregor et al. 2000). This does also apply for other autoimmune disorders such as insulin-dependent diabetes mellitus, where the concordance rate is around 40% (Hyttinen et al. 2003).

The prevalence of rheumatoid arthritis is very similar worldwide. However, numbers appear to be a bit higher in developed countries, with a prevalence of 0.5–1.0%, whereas in underdeveloped countries this prevalence ranges from 0.1 to 0.5% (Alamanos and

Correspondence: F. A. C. Rocha, Faculty of Medicine—Federal University of Ceará, Rua Dr José Lourenço, 1930—Aldeota, CEP 60115-281 Fortaleza, CE, Brazil. Tel: 55 85 4009 8243. Fax: 55 85 3244 6215. E-mail: arocha@ufc.br

ISSN 1740-2522 print/ISSN 1740-2530 online © 2006 Taylor & Francis
DOI: 10.1080/17402520600876895

Drosos 2005). However, the scarcity of well conducted epidemiological studies in the later compromise a more strict appreciation of these numbers.

### From São Paulo to Fortaleza

Socioeconomic disparities should influence the prevalence and the clinical picture of diseases worldwide. Recent data have emphasized that delay in diagnosis, difficulties in the access to a specialist, poor compliance to treatment and failure and delay in the indication of disease-modifying compounds have a profound impact in the evolution of autoimmune diseases. This appears to be particularly true for systemic lupus erythematosus and rheumatoid arthritis.

We work in the northeast of Brazil, in the state of Ceará. The city—Fortaleza—is located around 4°S latitude, by the Atlantic coast with around 2,500,000 inhabitants including the metropolitan area. Temperatures are stable around the year, varying from 22 to 32°C, and there is a low rainfall index. It is one of the poorest areas of the country, with a mean annual income rate of less than 2000 US$ per capita (IBGE-Brasil 2000). The health system in Fortaleza is a reference for the whole state of Ceará. This means that around 7,000,000 people, the population of the state, seek specialized medical care in Fortaleza.

After completing postgraduation in São Paulo, that is located 3000 km south from Fortaleza, and a postdoctoral fellowship in Sherbrooke-Québec, we moved back to Fortaleza to teach and practice rheumatology. During the first 3 years of rheumatology practice, I realized that many patients with inflammatory axial disease could be best classified as having undifferentiated spondyloarthropathies. This is to say that despite presenting axial and peripheral inflammatory pain, the vast majority of the patients had normal X-rays, even after 20 years of symptomatology. In addition, the patients with rheumatoid arthritis did not seem to be more severe as compared to my experience in São Paulo. This was very intriguing to me, if we consider that São Paulo is a developed state. My belief was that better education and higher lifestyle should positively influence the outcome of these disorders.

We have just started an epidemiologic survey in order to quantitatively assess our experience. However, data from the literature support our impression that these people, with a low socioeconomic status, poor access to the health system as well to medications, do not behave worse with regard to severity of inflammatory arthropathies, as might be anticipated. A recent publication from our region showed that people with systemic lupus erythematosus in the neighbor state of Rio Grande do Norte displayed similar damage, measured by the SLICC criteria, as compared to series reported from developed countries (Vilar et al. 2005). Except for more severe skin lesions, the clinical picture of these chronic patients was very similar to the casuistic reported in developed countries. We should stress that the high index of insolation as well as inadequate use of sun protectors should probably be the reason for this increased damage to the skin.

What could possibly be different in our environment that protected our patients from the expected crippling destiny of rheumatoid arthritis? We will try to present data that the answer may be "floating in the gut."

### In the gut

The Brazilian northeast is a highly endemic region of helminthiasis. Actually, our numbers of parasitic infestations are not known. However, the prevalence is so high that we usually treat patients, especially children, with symptoms that could be attributed to helminthiasis, without relying on stool examination. Investigation of inflammatory bowel diseases usually waits for the result of anti-helminthics and anti-protozoan medications in our daily practice. Chronic phases of schistosomiasis still occur, sometimes with the full-blown picture of hepatosplenomegaly.

Data from the literature have long supported a role for helminths in the modulation of inflammation. This subject has been elegantly explored in a recent review (Dunne and Cooke 2005). Particularly, schistosoma worms or its components were shown to ameliorate experimentally induced diseases as the experimental allergic encephalomyelitis, diabetes, and thyroiditis. *Trypanosoma brucei* was shown to inhibit the development of collagen-induced arthritis (Reviewed by Dunne and Cooke 2005). Perhaps more significant, inflammatory bowel disease in humans, that share some pathogenetic mechanism with inflammatory arthropathies, has been successfully treated by the oral administration of *Thichuris suis* eggs (Summers et al. 2005).

Considering the above, our hypothesis is that the similar or even lower (still to be proven) disease severity of the autoimmune rheumatic diseases in the northeast of Brazil is at least partially linked to endemic worm's infestations.

Previous studies from a group that we collaborate with have shown anti-inflammatory properties of an extract obtained from *Ascaris suum* (Soares et al. 1992). They have looked to immunoglobulin production, showing a decrease in both IgG and IgE levels after the administration of the *A. suum* extract to mice and rats. More recently, they are working on the modulation of experimental asthma, exploring the properties of *A. suum* components in the modulation of allergic inflammation (Itami et al. 2005).

### Zymosan-induced arthritis

The zymosan-induced arthritis model is characterized, in its acute phase, by a pronounced cellular infiltration into the joint space, with predominance of neutrophils. After 24 h, the cell influx subsides and there is a switch where mononuclear cells become predominant. After a single joint injection of 1 mg zymosan, the arthritis progresses and, in the chronic phase, between 7 and 14 days, severe destruction of the cartilage appears (Rocha et al. 1999). We have been working in this model in the past 15 years. Using the test for articular incapacitation for rats, we standardized the acute joint hyperalgesia that happens in the zymosan arthritis (Rocha et al. 1999). Further, we also developed a method to directly quantitate joint damage, by measuring the glycosaminoglycan content of the articular cartilage (Bezerra et al. 2004). Therefore, we are currently using the zymosan arthritis model as a means of studying inflammatory phenomena coupled to functional (pain) and structural damage parameters.

### *Ascaris suum* and experimental arthritis

During the II Latin–American Congress of Autoimmunity, held in Rio de Janeiro, Brazil, in April 2006, we presented preliminary data obtained from our group, where we evaluated the effect of the administration of an extract obtained from adult *A. suum* worms in the zymosan-induced arthritis model.

Our data revealed that parenteral, as well as oral administration of the *A. suum* extract to rats subjected to zymosan arthritis produced a dose-dependent significant reduction of the acute cell infiltration into the joint cavities. Additionally, joint hyperalgesia was also significantly inhibited, as compared to vehicle-treated rats. Similar data were obtained in mice subjected to zymosan-arthritis. Moreover, the chronic synovitis, evaluated by histopathology, was clearly abrogated, with a scant presence of lymphocytes and macrophages in the synovia of the animals that received the *A. suum* extract prior to the intra-articular injection of the zymosan. Finally, assessment of articular cartilage damage, by measurement of its glycosaminoglycan content, revealed that in the rats subjected to zymosan arthritis that received the *A. suum* extract there was a prevention of the glycosaminoglycan loss, as compared to vehicle-treated animals. In an attempt to explore the mechanisms involved in this protective effect, we obtained that the levels of interleukin-1$\beta$, but not those of tumor necrosis factor-$\alpha$, measured in the joint cavities, were significantly reduced in the group of animals that received the *A. suum* extract, as compared to vehicle-treated animals.

In summary, our data provide evidence that a helminth extract provides anti-inflammatory effects in an experimental arthritis model. The fact that the extract was equally effective regardless of being injected parenterally or orally suggests that it has pathophysiological relevance. Further, the phenomenon is not species-dependent, as it appeared in rats and mice and is associated to alteration in the local release of cytokines, in the joint milieu. We should also stress that the benefit occurred both at a functional and structural level, since the joint hyperalgesia, as well as the articular cartilage damage, were significantly reduced.

### Concluding remarks

Considering these data, coupled to the epidemiological aspects mentioned above, we sustain our proposal that the clinical picture of autoimmune diseases, at least the autoimmune rheumatic diseases, in humans, is probably affected by the exposure of these patients to helminths. The present data were obtained with a nematode, i.e. *A. suum*, but we would not be surprised if other parasites, as well as protozoa, may probably affect the evolution of autoimmune diseases. The implications of these data to our understanding of the immunoinflammatory regulation of autoimmune diseases can be clearly visualized. However, we also anticipate that therapeutic strategies may be generated from the better understanding of these pathways.

### References

Alamanos Y, Drosos AA. 2005. Epidemiology of adult rheumatoid arthritis. Autoimmun Rev 4(3):130–136.

Bezerra MM, Brain SD, Greenacre S, Jerônimo SMB, De Melo LB, Keeble J, Rocha FAC. 2004. Reactive nitrogen species scavenging, rather than nitric oxide inhibition, protects from articular cartilage damage in rat zymosan-induced arthrithis. Br J Pharmacol 141(1):172–182.

Dunne DW, Cooke A. 2005. A worm's eye view of the immune system: Consequences for evolution of human autoimmune diseases. Nat Rev Immunol 5:420–426.

Hyttinen V, Kaprio J, Kinnunen L, Koshenvuo M, Tuomilehto J. 2003. Genetic liability of type I diabetes and the onset age among 22,650 young Finnish twin pairs: A nationwide follow-up study. Diabetes 52:1052–1055.

IBGE-Brasil. Instituto Brasileiro de Geografia e Estatística, Censo 2000.

Itami DM, Oshiro TM, Araujo CA, Perini A, Martins MA, Macedo MS, Macedo-Soares MF. 2005. Modulation of murine experimental asthma by *Ascaris suum* components. Clin Exp Allergy 35(7):873–879.

Khan MA, Ball EJ. 2002. Genetic aspects of ankylosing spondylitis. Best Pract Res Clin Rheumatol 16(4):675–690.

MacGregor AJ, et al. 2000. Characterizing the quantitative genetic contribution to rheumatoid arthritis using data from twins. Arthritis Rheum 43:30–37.

Rashid T, Ebringer A, Wilson C, Bansal S, Paimela L, Binder A. 2006. The potential use of antibacterial peptide antibody indices in the diagnosis of rheumatoid arthritis and ankylosing spondylitis. J Clin Rheumatol 12(1):11–16.

Rocha FAC, Aragão Jr., AGM, Oliveira RC, Pompeu MML, Vale MR, Ribeiro RA. 1999. Periarthritis promotes gait disturbance in zymosan—induced arthritis in rats. Inflamm Res 48:485–490.

Soares MF, Mota I, Macedo MS. 1992. Isolation of *A. suum* components which suppress IgE antibody responses. Int Arch Allergy Appl Immunol 97(1):37–43.

Summers RW, Urban Jr., JF, Elliott DE, Thompson R, Weinstock JV. 2005. *Trichuris suis* therapy in Crohn's disease. Gut 54: 87–90.

Toivanen P. 2003. Normal inte stinal microbiota in the aetiopathogenesis of rheumatoid arthritis. Ann Rheum Dis 62:807–811.

Vilar MJ, Bezerra EL, Sato EI. 2005. Skin is the most frequently damaged system in recent-onset systemic lupus erythematosus in a tropical region. Clin Rheumatol August 24(4):377–380.

